# Medication, Nutrition, and Hygiene in COVID‐19 Prevention and Treatment: A Comprehensive Narrative Review

**DOI:** 10.1002/gch2.202500223

**Published:** 2025-09-13

**Authors:** Wan‐Ting Kuo, I‐Hsiu Lai

**Affiliations:** ^1^ De‐An Clinic Tainan City 711 Taiwan; ^2^ Department of Pediatrics An Nan Hospital, China Medical University, Tainan, Taiwan No. 66, Sec. 2, Changhe Rd., Annan Dist. Tainan City 709 Taiwan

**Keywords:** COVID‐19, hygiene, nutrients, pharmacological treatment, SARS‐CoV‐2

## Abstract

Despite advances in Coronavirus Disease 2019 (COVID‐19) prevention and treatment, emerging variants and persistent challenges continue to affect global health. Studies are retrieved from PubMed using title‐based searches for COVID‐19, SARS‐CoV‐2, and related therapies from 2020 to 2025, focusing on randomized controlled trials, systematic reviews, and clinical guidelines. This review explores treatments, nutrients, and adjuvant therapies that support the immune system in fighting COVID‐19. It highlights the role of antiviral medications such as remdesivir, nirmatrelvir/ritonavir, and molnupiravir in reducing mortality and hospitalizations. Additionally, adjunctive therapies like corticosteroids, interleukin‐6 (IL‐6) inhibitors, Janus kinase (JAK) inhibitors, and N‐acetylcysteine (NAC) are discussed for their potential to modulate inflammation. Nutritional support, including omega‐3 fatty acids, vitamins D, C, and A, zinc, selenium, and probiotics, enhances immune function. Preventive measures, such as hygiene practices, wearing masks, and physical distancing, reduce transmission. An integrated approach that combines antiviral treatments with adjunctive therapies, prevention, and nutrition is crucial for improving outcomes.

## Graphical abstract

This narrative review presents evidence from 2020 to 2025 on antiviral medications, adjunctive therapies, and nutritional interventions for COVID‐19. It highlights the roles of remdesivir, monupiravir, nirmatrelvir/ritonavir, corticosteroids, vitamins, and hygiene practices, emphasizing an integrated approach that combines pharmacologic treatments, immune support, and preventive strategies to improve patients' outcomes and reduce disease severity and transmission.

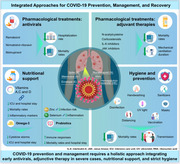

## 1 Introduction

Coronavirus Disease 2019 (COVID‐19), caused by the severe acute respiratory syndrome coronavirus 2 (SARS‐CoV‐2), has resulted in over 776 million reported cases and more than 7 million deaths globally since its emergence in December 2019, significantly impacting global health.^[^ ^1^ ^]^ Although it has now caused fewer casualties, new variants of SARS‐CoV‐2 continue to emerge.^[^ ^2^ ^]^ Therefore, it is essential to understand how viral infections can be prevented.

Clinical and demographic factors, such as advanced age, male sex, and smoking have been identified as significant risk factors for increased mortality among hospitalized COVID‐19 patients.^[^ ^3^ ^]^ In addition, the presence of chronic medical conditions such as chronic obstructive pulmonary disease (COPD), cardiovascular diseases (CVD), diabetes mellitus (DM), hypertension, cancer, acute kidney injury, obesity, and elevated D‐dimer levels has been strongly associated with poorer outcomes in COVID‐19. These comorbidities significantly increase the risk of severe disease progression and complications.^[^ ^3^ ^]^ These underlying conditions exacerbate disease severity and contribute to a higher likelihood of complications.

Selenium deficiency is a factor that exacerbates COVID‐19 severity.^[^ ^4^ ^]^ Nutritional support has been demonstrated to enhances immune function and facilitate recovery.^[^ ^5^, ^6^, ^7^ ^]^ Omega‐3 polyunsaturated fatty acids (PUFAs), vitamin D, and combined vitamin supplementation have shown promise in modulating immune responses. These nutrients may help mitigate severe inflammatory reactions, including cytokine storms, which are commonly observed in critical COVID‐19 cases.^[^ ^6^, ^8^, ^9^, ^10^ ^]^

Despite substantial advancements in COVID‐19 prevention and treatment, the disease remains a persistent global challenge, underscoring the need for effective strategies to curb disease progression, reduce hospitalization rates, and mitigate the risk of long COVID, an emerging chronic condition.^[^ ^11^ ^]^ Anti‐viral agents play an important role today; however, because of their high cost, their prescription is restricted to individuals with specific health conditions or older adults in most countries.^[^ ^12^ ^]^ Therefore, developing affordable, accessible, and effective alternatives is essential. A multifaceted approach is the key to enhancing immune function, minimizing transmission, and reducing disease severity for the effective COVID‐19 management. This review aimed to evaluate the current evidence on pharmacological treatments, nutritional interventions, and hygiene practices for the prevention and management of COVID‐19.

## 2 Materials and Methods

This narrative review explores the nutrients and treatments that may support the immune system in combating COVID‐19, along with hygiene practices that help prevent infection. To identify relevant studies, a comprehensive search was conducted in PubMed for articles published from 2020 to 2025, using the following search terms: “COVID‐19,” “SARS‐CoV‐2,” and “acute respiratory distress syndrome” in combination with “treatment,” “nutrition,” “vitamins,” “micronutrients,” “hygiene,” “sanitization,” “prevention,” “ immune,” “ risk factors,” “ pathogenesis,” “ symtoms,” “ mortality,” and “ complications.” Additional relevant studies were manually selected by reviewing the references of the articles identified in the initial search based on their clinical importance, necessary information, relevance, sample size, date, citation, and impact factors. Priority was given to randomized controlled trials (RCTs), systematic reviews, and clinical guidelines according to their clinical importance, necessary information, date, sample size, citations, and impact factors.

## 3 Pathogenesis of COVID‐19 and Immune Response

COVID‐19 is a respiratory disease caused by the novel coronavirus SARS‐CoV‐2, which can lead to complications in multiple organs, causing severe health outcomes. More than 200 viruses can cause common cold.^[^ ^13^ ^]^ Coronaviruses are a large family of pathogens; some often cause the common colds, whereas others have led to severe outbreaks, such as SARS, MERS, and COVID‐19. They evolve rapidly through gene mutations and recombination. According to Centers for Disease Control and Prevention (CDC), in the United States, respiratory viruses cause millions of illnesses, hundreds of thousands of hospitalizations, and thousands of deaths each year.^[^ ^14^ ^]^ Coronaviruses account for 15–30% of upper respiratory tract infections.^[^ ^15^ ^]^

Coronaviruses are single‐stranded positive‐sense RNA viruses with envelopes. There are five structural proteins: Spike protein (S), Membrane glycoprotein (M), Nucleocapsid protein (N), Envelope protein (E) and Hemagglutinin‐esterase protein (HE) in beta‐coronavirus. Three novel beta‐coronaviruses have caused outbreaks with high mortality rates Severe Acute Respiratory Syndrome (SARS‐CoV), Middle East Respiratory Syndrome (MERS‐CoV), and SARS‐CoV‐2.The pulmonary pathology of COVID‐19 involves alveolar damage, reactively hyperplastic pneumocytes, inflammatory cell infiltration as well as intravascular thrombosis.^[^ ^16^ ^]^

The membrane glycoproteins (M) facilitate viral assembly by adapting a portion of the membrane and capturing structural proteins at the budding site. The spike protein (S) mediates receptor binding and membrane fusion, while the envelope protein (E) aids in virus budding.^[^ ^17^ ^]^ The receptor‐binding domain (RBD) on the spike protein recognizes the host‐cell angiotensin‐converting enzyme 2 (ACE2) as entry receptor and initiates the first step of infection.^[^ ^18^ ^]^ In SARS‐CoV‐2, hemagglutinin‐esterase (HE) functions as a glycan‐binding lectin and receptor‐degrading enzyme.^[^ ^19^ ^]^ Additionally, the primary function of the nucleocapsid protein (N) is to package viral RNA into the capsid, and it can also bind to DNA in vitro.^[^ ^20^ ^]^ The virus enters human cells via binding the spike (S) protein to the angiotensin‐converting enzyme 2 (ACE2) as entry receptors, and also via priming spike protein by host cell serine protease TMPRSS2.^[^ ^21^ ^]^

ACE2 is highly expressed on ciliated and goblet cells in the lung, as well as type 2 alveolar cells which produce surfactant, moreover, ACE2 is also expressed on cardiac cells, vascular smooth muscle cells, vascular endothelium and intestinal epithelium.^[^ ^22^ ^]^ Viral clearance primarily depends on type I interferon and its downstream signaling pathways, which reprogram the cell into an “antiviral state”.^[^ ^23^ ^]^ During viral invasion, pathogen‐associated molecular patterns (PAMPs) are recognized by endosomal RNA receptors, TLR3 and TLR7 and cytosolic RNA sensor, RIG‐I/MDA5. This recognition activate NF‐kB and IRF3, and these transcription factors induce the upregulation of type I interferon, which activates JAK‐STAT pathway, and innate pro‐inflammatory cytokines, such as interleukin‐1 (IL‐1), interleukin‐6 (IL‐6), and tumor necrosis factor‐alpha (TNF‐α).^[^ ^24^ ^]^

Lymphopenia and cytokine storm may play critical roles in the pathogenesis of COVID‐19. This excessive immune response can trigger viral sepsis and inflammation‐induced lung injury, potentially leading to complications, such as pneumonitis, acute respiratory distress syndrome (ARDS), respiratory failure, shock, organ failure, and even death.^[^ ^24^ ^]^

CD4^+^ T lymphocytes (T helper cells) stimulate B lymphocytes to produce antibodies, whereas CD8^+^ T cells eliminate virus‐infected cells. Additionally, T helper cells release proinflammatory cytokines to enhance immune defense. White blood count, platelets decrease in COVID‐19 patients, but CD8 expression was higher ^[^ ^25^ ^]^ However, overproduction of pro‐inflammatory cytokines, reactive oxygen and nitrogen species, and excessive recruitment of immune cells can lead to the destruction of normal tissues, permanent lung damage, organ failure, and even death.^[^ ^26^ ^]^

In patients with SARS‐CoV‐2‐induced acute respiratory distress syndrome (ARDS), the immune system becomes hyperactivated, triggering a cytokine storm characterized by elevated levels of pro‐inflammatory cytokines such as IL‐2, IL‐6, IL‐7, IL‐22, CXCL10, and TNF‐α.^[^ ^27^ ^]^ Patients admitted to the intensive care unit (ICU) had significantly higher levels of IL‐2, IL‐7, IL‐10, G‐CSF, IP‐10, MCP‐1, MIP‐1A, and TNF‐α compared to non‐ICU patients.^[^ ^28^ ^]^ Among these, IL‐6, IL‐1, and TNF‐α are key cytokines responsible for driving cytokine storm.^[^ ^29^ ^]^ During the resolution phase of the disease, T cell counts recover, while levels of IL‐6, IL‐10, and TNF‐α decline.^[^ ^30^ ^]^

## 4 The Symptoms of COVID‐19 and Inflammatory Markers

Patients with COVID‐19 present with a wide range of symptoms, the most common being fever (16.3–91.0%, pooled prevalence: 64.6%), cough (30.0–72.2%, pooled prevalence: 53.6%), muscle soreness (3.0–44.0%, pooled prevalence: 18.7%), and fatigue (3.3–58.5%, pooled prevalence: 29.4%).^[^ ^31^ ^]^ Another study reported that cough (67.8%), fatigue (38.1%), sputum production (33.7%), and shortness of breath (18.7%) were the most frequently observed symptoms. Additional symptoms included myalgia (14.9%), sore throat (13.9%), headache (13.6%), chills (11.5%), nausea or vomiting (5%), nasal congestion (4.8%), and diarrhea (3.8%). Fever was detected in 43.8% of patients upon admission, but developed in 88.7% of patients during hospitalization.^[^ ^32^ ^]^ Although dyspnea has been identified as a predictor of higher mortality, fever has not.^[^ ^33^ ^]^ Additionally, alterations in smell or taste appear to be more prevalent in women than in men.^[^ ^34^ ^]^ Many patients with the Omicron variant present with acute odynophagia, severe sore throat, and fever, indicating COVID‐19‐associated acute laryngotracheitis or pharyngitis.^[^ ^35^ ^]^ Weakness is also a frequently observed symptom in critically ill COVID‐19 patients.^[^ ^36^ ^]^

The median incubation period for COVID‐19 was 4 days (interquartile range, 2–7 days). On admission, the common hematological abnormalities included lymphopenia (83.2%), thrombocytopenia (36.2%), and leukopenia (33.7%). Additionally, elevated levels of C‐reactive protein (CRP) (≥10 mg L^−1^) (60.7%), lactate dehydrogenase (LDH) (41%), D‐dimer (46.4%), and aspartate aminotransferase (AST) (22.2%) were frequently observed.^[^ ^32^ ^]^ In fatal cases, the platelet count and plasma albumin levels decreased significantly, while CRP, AST, serum creatinine, IL‐6, and LDH levels increased significantly. Abnormally high creatine kinase levels were also noted.^[^ ^37^ ^]^

## 5 The Complications of COVID‐19 and Immune Evasion

ARDS is one of the most severe complications of COVID‐19, often leading to respiratory failure and the need for mechanical ventilation,^[^ ^32^ ^]^ In a cohort of 72314 COVID‐19 patients, 81% experienced mild disease, 14% developed severe disease, and 5% progressed to critical illness characterized by respiratory failure, septic shock, or multiple organ failure with limb ischemia.^[^ ^38^ ^]^ Evidence shows that COVID‐19 affects multiple systems, including the neurological, cardiovascular, digestive, renal, endocrine, and dermatologic.^[^ ^39^ ^]^ Sepsis, often linked to a cytokine storm, may progress to septic shock, multiorgan failure, and high mortality rate.^[^ ^24^ ^]^ Some patients develop long COVID, which is characterized by persistent fatigue, respiratory difficulties, brain fog, and other lingering symptoms.^[^ ^11^ ^]^

A meta‐analysis found that COVID‐19 mortality was significantly associated with ARDS (odds ratio [OR] = 100.36), shock (OR = 96.60), secondary infection (OR = 30.25), acute heart injury (OR = 27.73), acute liver injury (OR = 3.15), and acute renal failure (OR = 28.11) compared to survivors, in mortality case, low platelet count, increased CRP, LDH and IL‐6 were also noted.^[^ ^37^ ^]^ A study of 184 ICU patients with COVID‐19 reported a 31% incidence of thrombotic complications, with pulmonary embolism being the most common.^[^ ^40^ ^]^ Puntmann et al. found that cardiac MRI revealed cardiac involvement in 78% of patients and ongoing myocardial inflammation in 60% of 100 patients at a COVID‐19 testing center.^[^ ^41^ ^]^

The mortality rate among COVID‐19 patients with ARDS is 45%, and ARDS occurs in 90% of fatal COVID‐19 cases. Some studies have estimated that 8–19% of COVID‐19 patients develop ARDS.^[^ ^16^ ^]^ A cohort study of 8625 hospitalized COVID‐19 patients reported an unadjusted 30‐day mortality rate of 5.70%, while among 2647 hospitalized influenza patients during the fall‐winter season of 2023–2024, the unadjusted 30‐day mortality rate was 3.04%.^[^ ^42^ ^]^ The complications of COVID‐19 stem from dysregulated immune responses, such as cytokine storm, whereas immune evasion involves mostly inhibition of innate immune responses, notably type I interferon recognition and signaling, as for adaptive immune responses, it involves downregulation of antigen presentation via MHC class I and MHC class II.^[^ ^24^ ^]^ Early intervention is crucial to mitigate life‐threatening complications.

Recent research has shown that influenza and COVID‐19 infections can reactivate dormant disseminated breast cancer cells (DCCs) in the lungs, leading to their proliferation and metastasis in mice within two weeks of infection. This process is initiated by interleukin‐6 and sustained by CD4⁺ T cells, which suppress the activation and cytotoxic function of CD8⁺ T cells. These findings are also consistent with observational data from human studies, including all cancer types in the UK Biobank and breast cancer data from Flatiron Health.^[^ ^43^ ^]^

## 6 Pharmacological Treatment for COVID‐19

### 6.1 Primary Antiviral Agents for COVID‐19 Treatment

**Table** **1** presents the key findings from recent studies on primary antiviral agents for COVID‐19, emphasizing their impact on clinical outcomes, particularly when administered early. Antiviral treatments such as remdesivir, nirmatrelvir/ritonavir (Paxlovid), and molnupiravir have demonstrated significant efficacy in reducing mortality, hospitalization, and ICU admissions while expediting viral clearance.^[^ ^44^, ^45^, ^46^ ^]^ Antiviral agents significantly improve clinical outcomes, particularly in high‐risk groups like the unvaccinated, by reducing disease progression.^[^ ^44^, ^46^ ^]^

**Table 1:** Summary of evidence on antiviral therapy or primary antiviral agents for COVID‐19 treatment.

Drug	Primary mechanism	Relevance to COVID‐19^a)^	Refs.
Remdesivir	Nucleoside analogues	Reduces mortality rate Shortens the time to recovery Decreases hospitalization rate	[45, 47]
Nirmatrelvir–ritonavir	Protease inhibitors	Decreases mortality and hospitalization rate Accelerates viral clearance Lowers all‐cause mortality risk	[46, 48, 49]
Molnupiravir	RNA polymerase inhibitor	Decreases mortality risk Reduces the risk of hospitalization Accelerates viral load decline but is linked to higher viral persistence, lower anti‐spike antibody titers, and higher transition mutations	[44, 48, 50]

^a)^ Data obtained from randomized clinical trials, systematic reviews, and meta‐analyses of clinical trials provide robust evidence.

Clinical evidence has underscored the benefits of remdesivir in COVID‐19 patients. In a double‐blind RCT of 1062 hospitalized patients, remdesivir (10‐day regimen) significantly shortened recovery time, improved clinical outcomes, and showed a trend toward lower mortality (11.4% vs 15.2% by day 29) with fewer serious adverse events than placebo.^[^ ^45^ ^]^ Pasquini et al. reported that in some ICUs, intubated patients had a mortality rate exceeding 80%. However, among 51 ICU patients in Italy with respiratory failure requiring intubation, mortality decreased from 92% in the control group to 56% in the remdesivir group.^[^ ^47^ ^]^

A meta‐analysis of 23 studies encompassing 314353 patients evaluated the efficacy and safety of nirmatelvir/ritonavir (Paxlovid) for COVID‐19. The analysis found that Paxlovid significantly reduced mortality (OR = 0.25; 95% confidence interval [CI]: 0.14–0.45), hospitalization (OR = 0.40; 95% CI: 0.24–0.69), hospitalization or death rate (OR = 0.17; 95% CI: 0.06–0.46), and PCR negative conversion time (mean difference [MD] = −2.46; 95% CI: −4.31 to −0.61).^[^ ^46^ ^]^

During the Omicron outbreak, an emulated trial of nirmatrelvir/ritonavir (Paxlovid) in 7119 hospitalized COVID‐19 patients (1813 received nirmatrelvir/ritonavir) showed a reduced risk of all‐cause mortality (HR 0.77, 95% CI: 0.66–0.90). Similarly, a Molnupiravir emulation study with 16495 hospitalized patients (2700 received Molnupiravir) demonstrated a lower mortality risk (HR = 0.87, 95% CI: 0.81–0.93).^[^ ^48^ ^]^ A retrospective study of 1354 unvaccinated moderate COVID‐19 patients with lung diseases found that nirmatrelvir/ritonavir (*n* = 738) was more effective than molnupiravir (*n* = 616) in reducing 90‐day mortality, with an adjusted hazard ratio (aHR) of 0.508 (95% CI: 0.314–0.822, *p* = 0.006).^[^ ^49^ ^]^

A double‐blind RCT assessed the efficacy and safety of molnupiravir in non‐hospitalized, unvaccinated adults with mild‐to‐moderate COVID‐19 and at least one risk factor for severe illness. A total of 1433 participants were randomly assigned to receive either 800 mg of molnupiravir or a placebo twice daily for five days. With hospitalization or death rates of 7.3% in the treatment group and 14.1% in the placebo group by day 29, molnupiravir decreased the risk of hospitalization or death.^[^ ^44^ ^]^

Another RCT investigated the effects of molnupiravir on viral clearance, antibody response, and mutagenesis in non‐hospitalized COVID‐19 patients. The participants (*n* = 577) were randomized to receive molnupiravir or usual care within five days of symptom onset. Molnupiravir accelerated the decline in viral load, but most patients still had detectable viruses by day 5. By Day 14, the molnupiravir group showed higher viral persistence (46/75 in the molnupiravir group compared to 21/70 in the Usual Care group), drug‐associated mutagenesis of ≥90% (32/46 in the Molnupiravir group) and lower anti‐SARS‐CoV‐2 spike protein antibody levels compared to usual care.^[^ ^50^ ^]^

Felsenstein et al. reported that combining protease inhibitors (lopinavir/ritonavir) with the nucleoside analog ribavirin reduced ARDS or mortality rates to 2.4% compared to 28.8% with ribavirin alone. Nucleoside analogs include favipiravir, galidesivir, ribavirin, and remdesivir.^[^ ^16^ ^]^

The effectiveness of antiviral therapy is greatest in the early stages of infection, when viral replication is at its peak and the adaptive immune response remains insufficient. Despite these benefits, there are some limitations. Nirmatrelvir/ritonavir does not appear to prevent viral rebound^[^ ^46^ ^]^ and its potential drug interactions require careful evaluation by clinicians before prescription.

In summary, antiviral agents effectively reduce mortality, hospitalization, and ICU admission when administered early. Remdesivir improves recovery and reduces mortality. While Paxlovid significantly lowers mortality in large studies, a list of medications or severe liver or renal function impairments are contraindications. Nirmatrelvir/ritonavir and molnupiravir benefit high‐risk individuals by improving outcomes, but, many patients are not eligible for anti‐viral agent prescriptions. However, challenges, such as viral rebound and potential adverse effects of molnupiravir, including increased viral persistence, remain unresolved. Early treatment is the key to maximizing efficacy.

### 6.2 Adjuvant Therapy

**Table** **2** summarizes the evidence on adjunctive therapy for COVID‐19. Emerging data suggest that N‐acetylcysteine (NAC) is a promising adjuvant therapy. Corticosteroids, IL‐6 inhibitors, and Janus kinase (JAK) inhibitors play pivotal roles in the management of severe to critical COVID‐19, particularly in patients experiencing dysregulated inflammatory responses.

**Table 2:** Summary of evidence on adjunctive therapies for COVID‐19 treatment.

Drug	Primary mechanism	Relevance to COVID‐19^a)^	Refs.
N‐acetylcysteine	Antioxidant and anti‐inflammatory	Reduces mortality rates, inflammatory markers, dyspnea, severe respiratory failure	[51, 52]
Dexamethasone	Corticosteroids	Reduces mechanical ventilation duration and increases ventilator‐free days, decreases all‐cause mortality rate^b)^, and the SOFA score	[53, 54]
Tocilizumab	Interleukin‐6 inhibitor	Reduces all‐cause mortality, risk of mechanical ventilation, ICU admission, and serious superinfections. Shows no mortality benefit in critically ill patients and patients on steroids	[55]
Sarilumab	IL‐6 inhibitor	Enhances clinical outcomes and reduces mortality rates	[56]
Baricitinib	JAK inhibitor	Reduces mortality rate and exhibits a safety profile	[57]
Tofacitinib	JAK inhibitor	Reduces the risk of death or respiratory failure	[58]
NRICM101, NRICM102	Traditional Chinese Medicine	Reduce mortality rate and prevent disease progression	[59]
Convalescent plasma		Reduces mortality rate and the risk of progression to hospitalization	[60]
Hydroxychloroquine/ chloroquine	Immunomodulatory agent	Hydroxychloroquine was associated with increased mortality, while chloroquine does not affect mortality, and may causes adverse effects, including arrhythmia	[61]
Azithromycin	Antibiotic	It does not improve clinical outcomes in patients with severe COVID‐19	[62]

^a)^ Data obtained from randomized clinical trials, systematic reviews, and meta‐analyses of clinical trials provide robust evidence.

^b)^ Including dexamethasone, hydrocortisone, or methylprednisolone. ICU, intensive care unit; JAK, Janus kinase 1/2 inhibitor; IL, Interleukin; SOFA, Sequential Organ Failure Assessment.

A randomized controlled trial compared NAC inhalation spray and routine treatment (*n* = 250) with routine treatment alone (*n* = 250) in COVID‐19 patients. The NAC group showed significantly lower mortality (3.2% vs 39.2%, *p* < 0.001), improved levels of inflammatory markers (CRP: 6 vs 11.5, *p* < 0.0001), and reduced aspartate aminotransferase (AST) levels (all p < 0.001). NAC also alleviated dyspnea (7.2% vs 19.2%, *p* = 0.005) and general weakness (3.2% vs 9.6%, *p* = 0.039) and was associated with a lower incidence of severe respiratory failure.^[^ ^51^ ^]^

In a trial of severely ill COVID‐19 patients, intravenous NAC treatment resulted in improved PaO_2_/FiO_2_ (*p* < 0.014) and reduced CRP (*p* < 0.001), D‐dimer (*p* < 0.042), and LDH (*p* < 0.001) levels after 3 days in the ICU compared to the control group. In addition, NAC mitigates the reduction in glutathione concentration.^[^ ^52^ ^]^

Corticosteroids have demonstrated significant benefits in patients with moderate to severe ARDS, including decreasing the need for mechanical ventilation, increasing ventilator‐free days, reducing secondary infections, and lowering Sequential Organ Failure Assessment (SOFA) scores.^[^ ^53^, ^54^ ^]^

A prospective meta‐analysis of seven randomized trials involving 1703 critically ill COVID‐19 patients assessed the effect of corticosteroids on 28‐day all‐cause mortality. The analysis found that corticosteroids significantly reduced mortality compared with usual care or placebo (OR = 0.66; *P* < 0.001). The study concluded that corticosteroids improve the survival of critically ill COVID‐19 patients.^[^ ^54^ ^]^ However, contrasting findings were reported in another meta‐analysis of 43 studies involving 96852 patients, which found that 2749 (14.2%) patients died in the intervention group (*n* = 19426; corticosteroid therapy) and 5459 (7.1%) died in the control group (*n* = 77426; standard treatment without corticosteroids).^[^ ^63^ ^]^ Furthermore, a retrospective study of 102 ICU patients revealed a 79.4% reduction in mortality in the pulse steroid therapy group compared to that in the high‐dose steroid group.^[^ ^64^ ^]^

IL‐6 inhibitors, including tocilizumab and sarilumab, have been shown to reduce mortality and improve clinical outcomes in COVID‐19 patients. These agents also reduce the need for mechanical ventilation, ICU admission, and the incidence of severe secondary infections.^[^ ^55^, ^56^ ^]^ However, their efficacy appears to be limited in critically ill patients and when corticosteroids are widely used (>80% of the cases). Notably, IL‐6 inhibitors have demonstrated no significant increase in adverse events,^[^ ^55^ ^]^ yet their long‐term effects, efficacy across diverse healthcare settings, and impact on varying standards of care warrant further investigation.^[^ ^56^ ^]^

Among the JAK inhibitors, baricitinib (a JAK 1/2 inhibitor) has been associated with reduced 28‐day mortality in hospitalized COVID‐19 patients.^[^ ^57^ ^]^ Although it did not significantly affect disease progression to severe illness, when combined with standard care (including dexamethasone), baricitinib exhibited a safety profile comparable to that of standard treatment alone.^[^ ^57^ ^]^ Similarly, tofacitinib, another JAK inhibitor, has demonstrated efficacy in reducing the risk of death or respiratory failure in hospitalized COVID‐19 patients.^[^ ^58^ ^]^

Two traditional medicine formulas from Taiwan, NRICM101 and NRICM102 may serve as potential alternative therapies for COVID‐19. In a multicenter retrospective study of 840 COVID‐19 patients across nine hospitals, seven mortality cases (5.69%) were reported in the NRICM102 group with usual care compared to 27 (21.95%) in the usual care group. None of the patients who received NRICM101 progressed to a more severe condition.^[^ ^59^ ^]^

Given the heightened risk of venous thromboembolism (VTE) in COVID‐19 patients, pharmacological thromboprophylaxis with heparin is recommended for hospitalized patients, with dosage adjustments based on disease severity, particularly for critically ill individuals.^[^ ^65^ ^]^

Several adjuvant therapies such as convalescent plasma therapy, hydroxychloroquine, chloroquine, and azithromycin have not consistently provided benefits for COVID‐19 patients. When administered early, convalescent plasma therapy has shown the potential to reduce disease progression, with relative risk reduction of 54%.^[^ ^60^ ^]^ However, its overall efficacy remains inconsistent, which limits its widespread use. Conversely, a large international meta‐analysis of randomized trials revealed that hydroxychloroquine was associated with increased mortality in COVID‐19 patients, while chloroquine provided no mortality benefit and was associated with adverse effects, including arrhythmias.^[^ ^61^ ^]^ Moreover, azithromycin, despite exhibiting immunomodulatory properties in non‐COVID‐19 ARDS, failed to improve clinical outcomes when used alongside standard COVID‐19 treatments.^[^ ^62^ ^]^

The emerging therapies for severe COVID‐19 include NAC, corticosteroids, IL‐6 inhibitors, and JAK inhibitors. NAC helps reduce mortality, inflammation, and respiratory failure, whereas corticosteroids and JAK inhibitors improve outcomes by reducing mortality and ventilator dependence. Heparin is recommended for thromboprophylaxis because of the risk of VTE. However, therapies such as convalescent plasma therapy are inconsistent, and hydroxychloroquine, chloroquine, and azithromycin lack clinical benefits. Therapeutic effectiveness varies according to the disease severity and treatment timing, highlighting the need for further research and patient‐specific protocols to optimize COVID‐19 management.

## 7 Nutritional Support in COVID‐19 Prevention and Recovery

**Table** **3** presents the current evidence from randomized controlled trials and systematic reviews, highlighting the potential of optimized nutritional supplementation to support immune function and improve outcomes in COVID‐19 patients. Adequate intake of key nutrients, including omega‐3 PUFAs and vitamins D, C, and A, as well as minerals like zinc and selenium, along with probiotics and proteins, plays a crucial role in enhancing immune responses, modulating inflammation, and reducing the risk of infection or severe disease progression.^[^ ^9^, ^10^ ^]^

**Table 3:** Summary of key vitamins, minerals, and nutrients for COVID‐19 prevention and recovery*, along with the main dietary sources of these nutrients.

Nutrient	Dietary sources	Relevance to COVID‐19^a)^	Refs.
Omega‐3 (EPA, DHA)	Fatty fish (salmon, mackerel), fish oil	Reduces cytokine storms, supports cardiovascular and respiratory health, and shortens ICU and hospital stays	[8]
Vitamin D	Fatty fish, fortified dairy, sunlight, egg yolks	Reduces severe outcomes and infections, mortality and ICU admission rates, fibrinogen levels	[9, 10]
Vitamin C	Citrus fruits, berries, peppers, leafy greens	Lowers hospital mortality and acute kidney injury without leading to adverse events	[66, 67]
Vitamin A	Carrots, sweet potatoes, spinach, liver	Reduces symptoms such as fever, body aches, fatigue, and inflammatory markers, including white blood cell count and C‐reactive protein	[5, 6, 67, 68, 69]
Zinc	Meat, shellfish, legumes, seeds	Decreases infection risk and shortens the duration of olfactory dysfunction	[70]
Selenium	Brazil nuts, seafood, eggs, whole grains	Enhances viral clearance and reduces inflammation	[4]
Probiotics	Yogurt, kefir, sauerkraut, kimchi	Improves gut microbiota balance, enhances immune responses, reduces mortality risk, length of hospital stays, the rate of non‐recovery, and the time to recovery	[7]
Protein	Meat, fish, eggs, beans, tofu	Essential for tissue repair, muscle recovery, and immune function, increases skeletal muscle index, improves hemoglobin levels, and reduces cases of sarcopenia	[71]

^a)^ Data obtained from randomized clinical trials, systematic reviews, and meta‐analyses of clinical trials, providing robust evidence.

DHA and EPA alleviate cytokine storms by producing anti‐inflammatory metabolites. Omega‐3 polyunsaturated fatty acids (ω‐3 PUFAs) are precursors to specialized pro‐resolution mediators (SPMs), which are essential for inflammation resolution.^[^ ^72^ ^]^ Supplementation with omega‐3 PUFAs in critically ill COVID‐19 patients receiving parenteral nutrition has been shown to improve clinical outcomes by reducing inflammatory markers such as CRP, IL‐6, and CXCL10 (an interferon‐γ inducible protein‐10). This supplementation has also been associated with shorter ICU and hospital stays.^[^ ^8^ ^]^ A meta‐analysis of 273 hospitalized COVID‐19 patients showed that supplementation with omega‐3 fatty acids was associated with a reduced risk of all‐cause mortality (RR = 0.76; 95% CI: 0.61, 0.93; *p* = 0.010).^[^ ^73^ ^]^

Vitamin D is crucial for immune regulation and adequate levels may reduce severe COVID‐19 outcomes. Vitamin D can reduce the risk of viral infections and mortality.^[^ ^74^ ^]^ Laaksi reported that 25‐hydroxyvitamin D plays an important role in innate immunity activation and mediates Toll‐like receptor stimulation and promotes the production of antimicrobial peptides (AMPs).^[^ ^75^ ^]^ AMPs, such as cathelicidin and defensins, protect the respiratory and gastrointestinal mucosa against infections by gram‐positive and gram‐negative bacteria, enveloped viruses, and fungi.^[^ ^76^ ^]^ Administering certain doses of cathelicidin can reduce influenza virus titers by 90% by directly acting on the influenza virion and inhibiting viral replication.^[^ ^77^ ^]^

A meta‐analysis of 4553 participants found that vitamin D supplementation reduced mortality (RR  =  0.72, 95% CI: 0.54–0.94, *p*  =  0.02) with continuous, multiple‐dosing (<100,000 IU totally over 14 days) more effective (RR = 0.53, 95% CI:0.34‐0.83, *p* = 0.006) than single‐dosing, ICU admission rates were also reduced, though no significant differences were observed in hospital stay length or intubation rates.^[^ ^9^ ^]^ In patients with vitamin D deficiency (25 (OH) D < 30 ng/mL), supplementation increases the proportion of SARS‐CoV‐2 RNA turned negative.^[^ ^10^ ^]^ Additionally, vitamin D supplementation significantly reduces fibrinogen levels, potentially lowering the clotting risk.^[^ ^10^ ^]^

It is widely accepted that high‐dose vitamin C can boost immunity against the common cold. Hemilä and Chalker reviewed 29 trials involving 11306 participants and found that vitamin C reduced the duration of common colds by 8% (3% to 12%) in adults and by 14% (7% to 21%) in children. In children, doses of 1000–2000 mg/day of vitamin C reduce the duration and severity of colds by 18%.^[^ ^78^ ^]^

Clinical studies have demonstrated that vitamin C supplementation can reduce hospital mortality in COVID‐19 patients (24.1% vs 33.9%; OR = 0.59; 95% CI: 0.37 to 0.95; *p* = 0.03) and lower the incidence of acute kidney injury (27.8% vs 45.0%; OR = 0.56; 95% CI: 0.40 to 0.78; *p* < 0.001).^[^ ^66^ ^]^ Although generally considered safe, vitamin C has been associated with prolonged ICU length of stay.^[^ ^66^ ^]^

Vitamin A Supports the Immune System against COVID‐19. It exhibits cytoprotective, antiviral, anti‐inflammatory, and immunomodulatory activity against SARS‐CoV.^[^ ^68^ ^]^ Additionally, vitamin A plays a key role in maintaining adequate levels of plasma natural killer cells, which have antiviral activity.^[^ ^79^ ^]^ Vitamin A supplementation (25000 IU/day for 10 days) alleviates symptoms such as fever, fatigue, and body aches in COVID‐19 outpatients.^[^ ^5^ ^]^ In critically ill patients, it reduces inflammatory markers such as white blood cell count and CRP levels.^[^ ^5^ ^]^

A prospective multicenter study found that lower vitamin A levels are associated with higher inflammatory marker levels (CRP, ferritin), reduced lymphocyte counts, and increased mortality in COVID‐19 patients.^[^ ^69^ ^]^ Critically ill patients had lower vitamin A levels than moderately ill ones, and low vitamin A (< 0.2 mg L^−1^) were associated with ARDS (OR = 5.54; *p* = 0.048) and mortality (OR = 5.21; *p* = 0.042).^[^ ^69^ ^]^ Yilmaz et al. also reported lower vitamins A (496 +/− 96 vs. 698 +/−93 ng/ml, *p* < 0.001) and vitamin C (2961 vs. 3953 ng/ml, *p* = 0.007) levels in COVID‐19 patients compared to healthy individuals.^[^ ^67^ ^]^

Zn supplementation enhances epithelial integrity, supports antiviral immunity, reduces the risk of hyperinflammation, and has antioxidant effects.^[^ ^26^ ^]^ Zn(2^+^) effectively inhibits RNA synthesis and the transcription complex of both SARS‐coronavirus and equine arteritis virus.^[^ ^80^ ^]^ In children, Zn supplementation led to a 15% reduction in pneumonia mortality.^[^ ^81^ ^]^ Zinc sulfate may reduce the duration of smell loss in COVID‐19 patients, though it does not affect the overall recovery time.^[^ ^70^ ^]^

Multimicronutrient supplements containing up to 200 µg per day of selenium (Se) have shown benefits in various viral infections, including coinfection with HIV and *Mycobacterium tuberculosis*.^[^ ^82^ ^]^ In a trial involving 80 hantavirus patients, 2 mg of selenite for 9 days reduced the mortality rate from 100% to 36.6%.^[^ ^83^ ^]^ Selenium levels were significantly higher in surviving COVID‐19 patients compared to non‐survivors (Selenium: 53.3 ± 16.2 vs 40.8 ± 8.1 µg L^−1^, selenoprotein P: 3.3 ± 1.3 vs 2.1 ± 0.9 mg L^−1^).^[^ ^4^ ^]^

A combination of vitamins A, B, C, D, and E significantly reduces inflammatory markers, disease severity, and clinical outcomes in critically ill COVID‐19 patients. These interventions lowered erythrocyte sedimentation rate, CRP, IL‐6, and TNF‐α, while also reducing prolonged hospitalization (>7 days), although no significant impact on mortality was observed.^[^ ^6^ ^]^

Probiotics and increased protein intake (1.2–1.5 g kg^−1^) enhance immune defenses and recovery in COVID‐19 patients, reducing mortality risk and hospital stays but not ICU admissions.^[^ ^7^ ^]^ In post‐infection cases, combining probiotics with diet therapy and physical training improves muscle mass, reduces sarcopenia, and increases hemoglobin levels.^[^ ^71^ ^]^

Other nutrients such as vitamins B6, B12, and E, as well as folate, iron, magnesium, and copper, may support the immune system. However, the findings from RCTs and systematic reviews among COVID‐19 patients have been inconsistent.

## 8 Hygiene and Prevention

To prevent the transmission of COVID‐19, it is essential to enhance disinfection practices, maintain social distancing, and use methods such as ultraviolet (UV) light, hypochlorous acid water, or 200 ppm bleach. Person‐to‐person transmission occurs via contact with infected secretions or airborne particles. This can occur when pathogens on our hands are transferred to our mucous membranes, as hands can carry millions of bacteria or other pathogens, whereas mucous membranes can only defend against ≈40% of them. Therefore, touching the nasal or oral mucosa with hands should be avoided.

Studies have shown that physical and social distancing help reduce SARS‐CoV‐2 transmission and COVID‐19 mortality, confirming its value as a public health measure. To maximize its effectiveness, it should be combined with mask use and good hand hygiene.^[^ ^84^ ^]^ Surface disinfection using alcohol, hypochlorite, hydrogen peroxide, peroxymonosulfate, and UV light effectively reduced SARS‐CoV‐2.^[^ ^85^ ^]^

Chin et al. reported that when the incubation temperature was increased to 70 °C, the SARS‐CoV‐2 virus was inactivated in less than 5 min.^[^ ^86^ ^]^ A UV‐C light dose (254 nm) of 3.7 mJ cm^−^^2^ can reduce the viral load by a factor of 2000 (>3‐log reduction) at concentrations similar to those observed in SARS‐CoV‐2 infection. A higher dose of 16.9 mJ cm^−^^2^ completely inactivates SARS‐CoV‐2 at all viral concentrations.^[^ ^87^ ^]^ Hypochlorous acid (HOCl), an endogenous substance which can also be produced by neutrophils, eosinophils, monocytes, and B lymphocytes, is a potent oxidant that is effective against a broad range of bacteria, fungi, and viruses.^[^ ^88^ ^]^

The Centers for Disease Control and Prevention (CDC) of the USA recommends alcohol‐based hand rubs (ABHR) containing at least 60% ethanol or 70% isopropanol for use in healthcare settings. However, handwashing with soap and water for at least 20 seconds remains essential when hands are visibly soiled, before meals, and after restroom use^[^ ^89^ ^]^ because alcohol is only effective against enveloped viruses instead of non‐enveloped viruses.

Vaccination has significantly reduced hospitalizations and deaths; however, coverage remains insufficient in many regions, leaving populations vulnerable to infection.^[^ ^44^ ^]^

This review had several strengths. First, it included the most up‐to‐date evidence (2020–2025) published in PubMed on pharmacological treatments, nutritional interventions, and hygiene practices for the prevention and management of COVID‐19. Second, this study mainly focused on randomized clinical trials, systematic reviews, and clinical guidelines, which strengthened the robustness of the findings. Third, this review critically evaluates nutrients and treatments that may enhance immune function, modulate inflammation, reduce ARDS, regulate the cytokine storm, and mitigate hospitalization and mortality rates in the fight against COVID‐19. Finally, this review highlights various hygiene practices and preventive measures to reduce viral transmission and mitigate disease severity.

Despite its contributions, the study has several limitations. First, the review mainly included PubMed studies, which may have introduced a publication bias. However, a focus on high‐quality randomized clinical trials and systematic reviews has helped mitigate this issue. Second, although varying methodologies and sample sizes may limit generalizability, the critical evaluation of these studies helps to identify patterns and guide future research.

## 9 Conclusion 

This narrative review emphasizes a comprehensive approach for COVID‐19 management that combines pharmacological treatment, nutritional support, and preventive measures. Early antiviral treatments reduce severity and hospitalization, whereas adjuvant therapies such as NAC and IL‐6 inhibitors improve outcomes in severe cases. Effectiveness depends on disease stage, comorbidities, and drug interactions. Nutritional support strengthens immunity and promotes recovery. Enhancing hygiene practices is crucial for reducing transmission. Ongoing monitoring of treatment safety, research on targeted nutrition, and early interventions are vital for optimizing outcomes.

## 10 Future Perspectives

Challenges such as emerging variants, long COVID, disparities in treatment access, and waking dormant disseminated cancer cells remain. We hope that there will be more and more anti‐vital agent inventions and productions, as well as other medications and adjuvant therapy trials. Moreover, governments or experts can be dedicated to educating ordinary people regarding the prevention of future emerging variant infections and how to take care of themselves during illness, thus lessening the burden on hospitals and national health insurance.

## Conflict of Interest

The authors declare no conflict of interest.

## Author Contributions

W.T.K. designed and conceived the study. W.T.K. and I.H.L. collected and analyzed the data. W.T.K. drafted the manuscript and interpreted the data. I.H.L. critically revised the manuscript. All authors have read and approved the final manuscript.
